# Levels of Physical Activity, Enjoyment, Self-Efficacy for Exercise, and Social Support Before and After Metabolic and Bariatric Surgery: a Longitudinal Prospective Observational Study

**DOI:** 10.1007/s11695-023-06887-7

**Published:** 2023-10-14

**Authors:** Annika Imhagen, Jan Karlsson, Emma Ohlsson-Nevo, Erik Stenberg, Stefan Jansson, Lars Hagberg

**Affiliations:** 1 University Health Care Research Center, Faculty of Medicine and Health, Örebro University, SE-701 82 Örebro, Sweden; 2 Department of Surgery, Faculty of Medicine and Health, Örebro University, SE-701 82 Örebro, Sweden; 3 School of Medical Sciences, Örebro University, SE-701 82 Örebro, Sweden

**Keywords:** Metabolic and bariatric surgery, Physical activity, Accelerometer, Mediators, Enjoyment, Self-efficacy, Social support

## Abstract

**Introduction:**

Physical activity (PA) after metabolic and bariatric surgery (MBS) can influence weight loss, health status, and quality of life. Known mediators to participate in PA are enjoyment, self-efficacy, and social support. Little is known about PA behavior in MBS individuals. The aim of this study was to explore levels of PA and the PA mediators enjoyment, self-efficacy, and social support before and after MBS and to investigate changes over time.

**Methods:**

Adults scheduled to undergo MBS were recruited from a Swedish university hospital. Accelerometer-measured and self-reported PA, body weight, and PA mediators were collected at baseline and at 12 to 18 months post-surgery.

**Results:**

Among 90 individuals included, 50 completed the follow-up assessment and had valid accelerometer data. Sedentary time (minutes/day) was unchanged, but sedentary time as percentage of wear time decreased significantly from 67.2% to 64.5% (p<0.05). Time spent in light PA and total PA increased significantly from 259.3 to 288.7 min/day (*p* < 0.05) and from 270.5 to 303.5 min/day (*p* < 0.01), respectively. Step counts increased significantly from 6013 to 7460 steps/day (*p* < 0.01). There was a significant increase in self-reported PA, enjoyment, self-efficacy for exercise, and positive social support from family. The increase in PA mediators did not lead to a significant change in time spent in moderate to vigorous PA.

**Conclusion:**

The increase in PA-mediators was not associated with an increase in moderate to vigorous PA, but the strengthened PA mediators suggest potential for an increase in moderate to vigorous PA in patients undergoing MBS.

**Graphical abstract:**

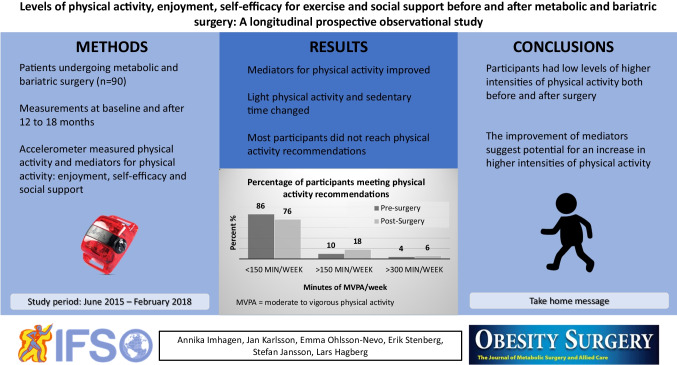

**Supplementary Information:**

The online version contains supplementary material available at 10.1007/s11695-023-06887-7.

## Introduction

Persons seeking metabolic and bariatric surgery (MBS) tend to spend most of their time in sedentary behaviors and perform low levels of physical activity (PA) [1, 2]. Participation in regular PA can be challenging because of both obesity- (e.g., physical limitations, negative body image) and non-obesity-related barriers (e.g., time constraints, low financial resources, weather, lack of motivation, lacking social support) [3, 4]. Several aspects including socio-demographic, biological, psychological, social, and environmental factors may influence the decision to perform PA and maintain it long-term [5].

PA after MBS is important because it contributes to greater weight loss, helps maintain a lower weight, and can reduce risk of diseases and improve quality of life [6–10]. World Health Organization (WHO) guidelines currently recommend at least 150–300 min/week of PA of moderate intensity, or 75–150 min/week of vigorous intensity, or an equivalent combination of both. The guidelines also state that sedentary time should be limited and replaced with PA of any intensity [11].

Weight loss after MBS can facilitate participation in PA; however, the literature on PA after MBS is conflicting. A meta-analysis from 2019 found no increase in objectively measured PA within 6 months post-surgery but found significant improvement after 6 months [12], while other studies report no increase [13, 14].

Psychological factors are central to the decision to perform PA; the main PA mediators are enjoyment, self-efficacy, and social support. Self-efficacy is a person’s confidence in their ability to perform a particular behavior [5]. Weight loss may affect PA mediators [2, 15, 16], but further studies are needed to evaluate determinants of PA behavior in individuals who have undergone MBS. The aim of this study was to explore levels of PA and the main PA mediators before and after MBS.

## Methods

### Participants

The study included adults ≥ 18 years who were scheduled to undergo primary Roux-en-Y gastric bypass (RYGB) or sleeve gastrectomy (SG) at a Swedish university hospital. Participants were recruited between June 2015 and February 2018. The indication for MBS was based on a modified version of the 1991 National Institutes of Health criteria, including body mass index (BMI) ≥ 35 with or without obesity-related comorbidity, previous weight loss attempts, mental stability, and no substance abuse ≥ 2 years before surgery [17]. In accordance with present guidelines, a preoperative weight loss of ≥ 5% of body weight was required [18]. Non-Swedish/English-speaking persons were excluded from the present study.

### Assessments

Measures of PA, self-efficacy for exercise, PA enjoyment, social support for exercise, and body weight were collected prior to surgery and at the1-year follow-up visit which, due to medical and practical reasons, took place at 12 to 18 months post-surgery. This is an appropriate time to evaluate lifestyle changes after a significant weight loss. Medical data and education level were retrieved from the Scandinavian Obesity Surgery Registry, a national quality and research registry [19].

### Assessments of Physical Activity

#### Accelerometer

PA was measured using an accelerometer (ActiGraph GT1M or wGT3X-BT, ActiGraph Inc., Pensacola, FL, USA). The accelerometer was sent to participants by mail, with instructions to wear it on an elastic belt on the back or hip during all waking hours for 7 consecutive days, except during showering and bathing. Participants were asked to keep an exercise diary, reporting when the accelerometer was put on and taken off, and type of PA performed. Data was reintegrated to 60-sec epochs (an epoch is a defined interval of movement data recorded by the accelerometer) and analyzed using ActiLife6 (ActiGraph 2012 ActiLife version 6.13.4; ActiGraph Inc.). Valid wear time was defined as ≥ 10 h/day for ≥ 3 days [20]. Non-wear time was defined according to Troiano as 60 min with no counts, allowing for up to 2 min with counts [21]. Counts are a result of summing accelerometer values into epoch “chunks.” The value of the counts varies depending on the frequency and intensity of the acceleration (ActiGraph Inc., Pensacola, FL, USA). Sedentary time was defined as < 100 counts per minute (cpm), light PA (LPA) as 101–3,207 cpm, and moderate to vigorous PA (MVPA) as > 3208 cpm [22].

#### Self-Reported Physical Activity

The Swedish National Board of Health and Welfare’s validated questions about the frequency of vigorous and moderate PA were used to measure self-reported PA [23]. The two questions are answered on a 6-point response scale: 0, < 30, 30–60, > 60–90, > 90–120, and > 120 min/week. A summary score is calculated by multiplying the answer to the first question by 2 (to account for a higher intensity) and adding the product to the answer to the second question. The summary score ranges from 3 (lowest level) to 18 (highest level) [23].

### Self-Assessments of Physical Activity Mediators

#### Enjoyment

Enjoyment was measured using the validated Physical Activity Enjoyment Scale (PACES) [24] modified by Motl et al. [25], consisting of 16 items rated on a 5-point scale. The total score ranges between 16 and 80, with higher scores indicating higher PA enjoyment [24, 26].

#### Self-Efficacy for Exercise

Self-efficacy was assessed using the validated Self-Efficacy for Exercise Scale (SEES) [27], which measures the ability to exercise 20 min three times a week based on perceived barriers to exercise. The SEES consists of nine items rated on a 10-point scale. A mean score is calculated (range 0–10), and higher scores indicate higher self-efficacy [28].

#### Social Support

Social support was assessed using the validated 13-item Social Support for Exercise Scale (SSES) [29], which is divided into a family and friends section, measured on a 5-point scale. Items measure social interactions that may be linked to exercise behavior (at least 20 min three times a week) during the past 3 months. The answers are summarized in three domains: family support–positive (range 11–55), family support–negative (negative comments about PA) (range 2–10), and friend support–positive (range 11–55).

### Statistical Analysis

Calculations based on a study assessing PA in women undergoing RYGB [30] indicate that a sample size of 100 completed participants would give a power of 94% to detect a 10% change in objectively measured sedentary time at *p* = 0.05. Categorical data was tested with the Chi-2 test. Means and standard deviations (SDs) were calculated. Normal distribution was evaluated graphically using histograms. Paired samples *t* tests were used to test for changes in PA and PA mediators over time. Effect size of within-group change was estimated by calculating the standardized response mean (SRM), i.e., the mean change divided by the SD of change. The SRM was evaluated according to standard criteria: < 0.20 = trivial, 0.20–0.49 = small, 0.50–0.79 = moderate, and ≥ 0.80 = large [31]. A *p*-value <0.05 was considered statistically significant. Analysis was performed using SPSS version 27 (IBM, Armonk, NY, USA).

## Results

### Participants

**Fig. 1:**
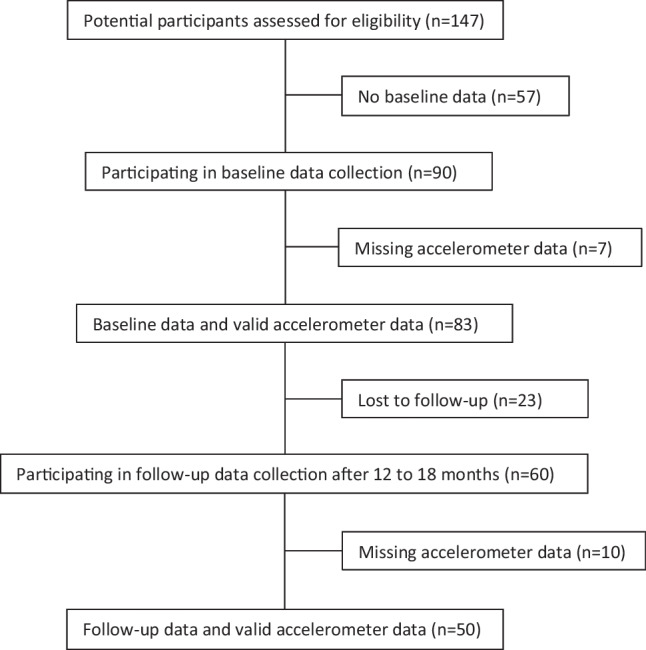
Flowchart showing participants

A total of 147 individuals provided consent, but 57 did not complete the baseline assessments (supplementary Figure 1), leaving a study sample of 90 participants. Of these, 60 completed the follow-up assessments, and 50/60 had valid accelerometer data (Fig. 1). Baseline characteristics of completers and dropouts are presented in Table 1. A higher proportion of non-completers were operated with SG. Mean (SD) percentage total body weight loss (%TWL) was 33.2 (7.9), and mean (SD) change in BMI was 13.8 (4.0).

**Table 1:** Baseline characteristics of completers and dropouts

	Completers (*n* = 60)	Dropouts (*n* = 30)	*p*
Age (years)*	42.4 (12.4)	39.2 (13.3)	0.27
Sex			
Female	46 (77%)	25 (83%)	0.47
Male	14 (23%)	5 (17%)	
Weight (kg)*	119.0 (20.8)	117.3 (24.4)	0.73
BMI (kg/m^2^)*	41.8 (5.9)	41.5 (6.4)	0.11
Level of education			
≤ 12 years of school	45 (75%)	24 (79%)	0.72
> 12 years of school	15 (25%)	6 (21%)	
Surgery method			
Gastric bypass	51 (85%)	20 (67%)	< 0.05
Gastric sleeve	9 (15%)	10 (33%)	

*Mean (SD)

### Physical Activity

**Fig. 2:**
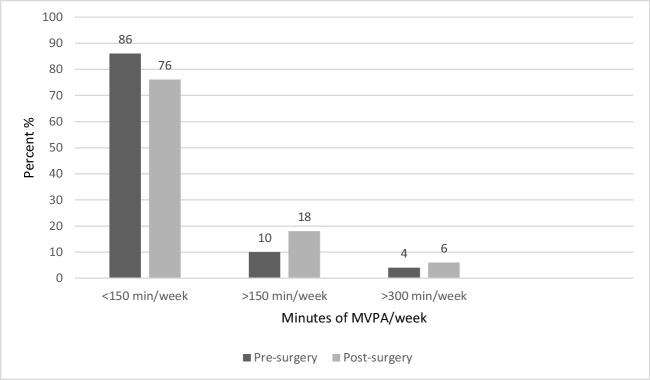
Percentage of participants meeting physical activity recommendations, pre- and post-surgery. MVPA, moderate to vigorous physical activity

Table 2 presents levels of sedentary time, LPA, MVPA, total PA, and step counts at baseline and follow-up. Mean valid accelerometer wear time, pre- and post-surgery, was 13.7 and 14.2 h/day, respectively (*p* < 0.05). The change in mean sedentary time (min/day) was not significant (*p* = 0.79); however, sedentary time as percentage of wear time decreased significantly from 67.2% pre-surgery to 64.5% post-surgery (*p* < 0.05). Participants increased their time spent in LPA from 259.3 to 288.7 min/day (*p* < 0.05). Moderate to vigorous PA was 11.2 min/day pre-surgery and 14.9 min/day post-surgery (*p* = 0.06). Total PA increased from 270.5 to 303.5 min/day (*p* < 0.01). Altogether, about 30 min/day of non-wear time was replaced with about 30 min/day of LPA. Steps increased from 6013 steps/day pre-surgery to 7460 steps/day post-surgery (*p* < 0.01). The summary score for self-reported PA increased from 7.9 to 10.5 (*p* < 0.001). Almost one-fourth of participants increased their MVPA by > 10 min/day post-surgery (Table 3), and 18% of participants reached PA recommendations of > 150 min of MVPA/week post-surgery, compared to 10% pre-surgery; 6% reached > 300 min of MVPA/week post-surgery compared to 4 % pre-surgery (Fig. 2).

**Table 2:** Self-reported and accelerometer-measured physical activity (PA) and mediators of PA before and after surgery

	n	Before surgery, mean (SD)	After surgery, mean (SD)	Difference, *p*-value	SRM
Self-reported PA	59	7.9 (3.4)	10.5 (4.2)	<0.001	0.62
Accelerometer-measured PA					
Valid wear time (hours/day)	50	13.7 (1.0)	14.2 (1.3)	< 0.05	0.32
Sedentary time	50				
min/day		550.0 (63.2)	547.0 (79.1)	0.79	0.04
% of wear time		67.2 (7.6)	64.5 (8.3)	< 0.05	0.31
LPA	50				
min/day		259.3 (68.3)	288.7 (78.9)	<0.05	0.35
% of wear time		31.5 (7.4)	33.8 (8.4)	0.07	0.27
MVPA	50				
min/day		11.2 (15.6)	14.9 (18.3)	0.06	0.27
% of wear time		1.4 (1.9)	1.8 (2.2)	0.12	0.23
Total PA (min/day)	50	270.5 (69.0)	303.5 (79.0)	< 0.01	0.40
Steps/day	47	6013 (2700)	7460 (3102)	< 0.01	0.45
Mediators of PA					
Enjoyment	55	54.8 (13.1)	65.3 (9.8)	<0.001	0.84
Self-efficacy	52	4.2 (2.4)	5.3 (2.4)	<0.01	0.39
Social support					
Family–positive	52	20.1 (9.5)	22.7 (10.2)	< 0.05	0.29
Family–negative	55	2.1 (0.5)	2.3 (1.0)	0.38	0.12
Friends–positive	54	21.9 (10.7)	21.9 (10.7)	1.0	0.00

*LPA* light physical activity, *MVPA* moderate to vigorous physical activity, *SD* standard deviation, *SRM* standardized response mean, where 0.2–0.49 = small, 0.5–0.79 = moderate, and 0.8+ = large effect. Steps/day: missing data from three participants at baseline due to technical problems. Self-reported PA score ranges from 3 to 18; higher scores indicate more time spent in PA during a week. Enjoyment scores ranges from 16 to 80; higher scores indicate higher enjoyment performing PA. Self-efficacy scores ranges from 0 to 10; higher scores indicate higher self-efficacy for PA. Social support: family–positive ranges from 11 to 55, a higher score indicating higher social support; family–negative ranges from 2 to 10 and higher scores indicate lower social support. Friends–positive ranges from 11 to 55

**Table 3:** Change in moderate to vigorous physical activity (MVPA) (min/day) post-surgery

MVPA change (min/day)	Number of participants (%)	
Decreased by > 20 min/day	2	4%
Decreased by > 10–20 min/day	3	6%
Decreased 1–10 min/day	7	14%
Decreased, total	**12**	**24%**
Unchanged	11	22%
Increased by 1–10 min/day	15	30%
Increased > 10–20 min/day	7	14%
Increased by > 20 min/day	5	10%
Increased, total	**27**	**54%**
Total	50	100%

Bold numbers are the total number / percentage of participants who decreased and increased their MVPA, respectively

### Mediators for Physical Activity

Post-surgery, there was an increase in PA enjoyment, from 54.8 to 65.3 (*p* < 0.001). Self-efficacy for exercise increased from 4.2 to 5.3 (*p* < 0.01). Positive social support from family for PA increased from 20.1 to 22.7 (*p* < 0.05). Negative social support from family and positive social support from friends remained unchanged (Table 2).

## Discussion

Light PA, total PA, steps/day, and self-reported PA all increased, but the results show generally low levels of higher PA intensities both before and after MBS. Sedentary time percentage of wear time increased significantly while mean sedentary time and MVPA (min/day) remained unchanged.

The PA mediator enjoyment, self-efficacy for exercise, and positive social support from family increased significantly post-surgery, in line with previous studies [2, 15, 32]. Weight loss can improve physical functioning and reduce pain and thus makes it easier to perform PA [12]. This may in turn enhance PA enjoyment, self-efficacy, and belief in own ability to perform PA. The effect size of the change in PA enjoyment was large (SRM = 0.84), which indicates that the positive experience of PA was considerably higher post-surgery. This is likely due to lower body weight and a reduction in obesity-related barriers to PA [33]. However, the effect size of change for self-efficacy and social support from family was small (SRM = 0.39 and 0.29), suggesting that these changes were less important than the change in enjoyment. With improved PA mediators, we suggest that there is potential for an increase in MVPA; however, to achieve this, additional interventions are needed and should be offered to patients undergoing MBS.

The increase in mediators for PA indicates a positive change in the participants’ perception of PA, which can be a first step towards a change in behaviors. However, it did not lead to a significant change in MVPA at group level, which has also been shown in previous studies [13, 14]. To accomplish an increase in higher intensities of PA, interventions should focus on habits. Habits develop when a behavior is repeated in a consistent context for an extended period of time [34]. If the experience of performed PA is satisfactory, there is an enhanced tendency to repeat the action. Over time cognitive shortcuts develop—a new habit is formed [35]. The challenge is to put habit theories into clinical practice to help individuals who have undergone MBS develop new PA habits.

The proportion of individuals meeting PA recommendations in a population may vary. A Swedish cohort study observed fivefold higher odds for high sedentary time and low MVPA in persons with BMI ≥ 35 compared to persons with BMI < 24.9 [36]. Most participants (82%) in our study did not reach the recommended PA levels [11]. On the group level, mean MVPA was 78.4 min/week pre-surgery and 104.3 min/week post-surgery. However, even if this change was not significant, the effect size suggests a small and possibly meaningful increase in MVPA. Almost 25% of participants increased their MVPA by at least 10 min/day. Additionally, 18% of the participants reached > 150 min of MVPA/week post-surgery, compared to 10% pre-surgery, which is consistent with a Norwegian study where 17.9% met the recommended MVPA level at 1-year post-surgery [37]. This increase may have a positive effect on the individual’s health status if PA is regularly performed at this level. Furthermore, WHO guidelines on PA state that health benefits can occur even with PA levels below recommendations [11].

Mean valid accelerometer wear time differed significantly pre- and post-surgery, and participants wore the accelerometer 30 min longer per day post-surgery. We do not know the reason for these differences, but a large weight loss may result in being out of bed for longer during the day. Another reason could be a better routine to wear the accelerometer at the follow-up. Longer wear time will likely result in longer sedentary time, as people in general spend more than half of their waking time sedentary [36]. When considering percentage of wear time, our results show a significant reduction in sedentary time post-surgery, in contrast to using actual time, which gave a non-significant result. However, the effect size of change was in the small range (SRM = 0.31), and it is somewhat unclear if the change is of clinical significance. Time spent in LPA and MVPA is probably not affected as much by wear time as is sedentary time. The most relevant results in our study are therefore the increase in actual time spent in LPA and MVPA and the decrease in sedentary time as percentage of wear time.

Persons with obesity often report that they are not used to performing PA and do not identify as someone who practices PA [32, 38], which can make it challenging to start or increase PA. Furthermore, non-obesity-related barriers to PA (e.g., lack of time, motivation, social support) may remain post-surgery [33].

Findings in the current and previous studies indicate that MBS is not sufficient to change a person’s PA habits. There is a need for further research to understand what motivates patients post-MBS to decrease sedentary time and perform PA of higher intensities and how best to support them. Previous studies show that this objective can be difficult to achieve. Neither group-based educational programs of four to ten sessions, nor a motivational individual PA intervention for 6 months resulted in increased PA [39–41]. An intervention focusing on mediators for PA, motivational theories, and how habits are formed may be more successful. Future research could test if internet-based cognitive behavioral therapy with additional support from health care professional could be appropriate for an intervention to increase PA after MBS. Although self-efficacy for exercise improved after MBS, the post-op scores are at a low level and additional treatment efforts may be directed specifically at improving self-efficacy.

### Strengths and Limitations

The major strengths of this study include the longitudinal design with objective monitoring of PA with accelerometers, as well as the investigation of mediators for PA. It is one of few studies using validated measures of enjoyment, self-efficacy for exercise, and social support in relation to PA post-MBS.

The power calculation indicated a sample size of 100 participants to achieve a power of 94%. However, the SD of sedentary time was lower in our study (63.2 min/day) than in the study the power calculation was derived from (141.5 min/day), and the power in our study was as strong as in the power calculation.

A major limitation of our study is the large dropout rate. At follow-up, 67% of the participants completed the questionnaires, but only 56% had also valid accelerometer data. It was a challenge to measure PA in individuals in free-living conditions. Despite intensive monitoring, several accelerometers were not returned, resulting in complete follow-up measures from 50 participants. Therefore, the results need to be interpreted with some caution. A higher proportion of dropouts were operated with SG; otherwise, we found no significant differences between completers and dropouts.

Our study population of 90 participants represents almost 20% of all persons who underwent MBS at the university hospital during the study period. Due to organizational reasons and periods of lack of personnel at the surgery unit, only 147 of 455 operated patients were asked for consent to participate in the study. It is possible that persons who were not asked or declined participation were less physically active, which may have biased our results.

We assessed outcomes 12 to 18 months after MBS, and participants may have experienced the “honeymoon effect” with major weight loss and maximum motivation at this point of time. Effects also need to be evaluated in the longer term to determine whether the changes are sustainable over time.

## Conclusion

Results in the present study show a significant increase in enjoyment of, and self-efficacy and social support for, PA. Light PA and total PA increased, and sedentary time as percentage of wear time decreased, but the increase in mediators for PA did not result in an increase in MVPA on group level.

### Supplementary Information

ESM 1 (DOCX 31 kb)
