# Supplementary material for: Levels of Physical Activity, Enjoyment, Self-Efficacy for Exercise, and Social Support Before and After Metabolic and Bariatric Surgery: a Longitudinal Prospective Observational Study
Source: Obes Surg. 2023 Oct 14;33(12):3899–906. doi: 10.1007/s11695-023-06887-7 (PMC10687134; doi:10.1007/s11695-023-06887-7)
Supplement: Supplementary file 1 — (DOCX 31 kb) [file 11695_2023_6887_MOESM1_ESM.docx]

**Supplementary Fig. 1**

Flowchart explaining reasons for eligible participants not participating in baseline collection.

Potential participants assessed for eligibility (n=147)

Declined to participate (n=10)

Surgery before baseline collection (n=14)

Surgery postponed (n=3)

Questionnaires and accelerometer were lost in the mail (n=5)

Participating in baseline data collection (n=90)

Did not participate in baseline collection for unclear reasons (n=25)
